# Qualitative insights into the experience of teaching shared decision making within adult education health literacy programmes for lower‐literacy learners

**DOI:** 10.1111/hex.12580

**Published:** 2017-07-05

**Authors:** Danielle M. Muscat, Suzanne Morony, Sian K. Smith, Heather L. Shepherd, Haryana M. Dhillon, Andrew Hayen, Lyndal Trevena, Karen Luxford, Don Nutbeam, Kirsten J. McCaffery

**Affiliations:** ^1^ University of Sydney School of Public Health Sydney NSW Australia; ^2^ University of Sydney Centre for Medical Psychology and Evidence‐based Decision‐making (CeMPED) Sydney NSW Australia; ^3^ Psychosocial Research Group Prince of Wales Clinical School Faculty of Medicine University of New South Wales Sydney NSW Australia; ^4^ University of Sydney Psycho‐oncology Co‐operative Research Group (PoCoG) School of Psychology Sydney NSW Australia; ^5^ University of Sydney School of Psychology Sydney NSW Australia; ^6^ University of Technology Sydney Faculty of Health Sydney NSW Australia; ^7^ Clinical Excellence Commission Sydney NSW Australia

**Keywords:** adult education, empowerment, health literacy, literacy, qualitative, shared decision making

## Abstract

**Background:**

Enhancing health literacy can play a major role in improving healthcare and health across the globe. To build higher‐order (communicative/critical) health literacy skills among socially disadvantaged Australians, we developed a novel shared decision making (SDM) training programme for adults with lower literacy. The programme was delivered by trained educators within an adult basic education health literacy course.

**Objective:**

To explore the experience of teaching SDM within a health literacy programme and investigate whether communicative/critical health literacy content meets learner needs and teaching and institutional objectives.

**Design and participants:**

Qualitative interview study with 11 educators who delivered the SDM programme. Transcripts were analysed using the Framework approach; a matrix‐based method of thematic analysis.

**Results:**

Teachers noted congruence in SDM content and the institutional commitment to learner empowerment in adult education. The SDM programme was seen to offer learners an alternative to their usual passive approach to healthcare decision making by raising awareness of the right to ask questions and consider alternative test/treatment options. Teachers valued a structured approach to training building on foundational skills, with language reinforcement and take‐home resources, but many noted the need for additional time to develop learner understanding and cover all aspects of SDM. Challenges for adult learners included SDM terminology, computational numerical risk tasks and understanding probability concepts.

**Discussion and conclusions:**

SDM programmes can be designed in a way that both supports teachers to deliver novel health literacy content and empowers learners. Collaboration between adult education and healthcare sectors can build health literacy capacity of those most in need.

## 1 INTRODUCTION

Given that lower health literacy is associated with poorer health outcomes^1^ and higher healthcare utilization and costs,^2^ improving health literacy is a policy initiative in most developed countries. Enhancing health literacy can play a major role in improving healthcare and health across the globe.^3^ Health literacy describes specific literacy skills needed to obtain, understand and use information to make decisions and take actions that will have an impact on health status.^4^ According to Nutbeam (2000), health literacy skills comprise three levels; functional, communicative and critical health literacy.^5^ Functional health literacy refers to the basic skills for obtaining health information; communicative and critical health literacy require more advanced skills to extract information, derive meaning from and critically evaluate health‐related material.^5^

Shared decision making (SDM) may be viewed within Nutbeam's conceptual framework as corresponding to communicative and critical health literacy. Specifically, SDM occurs when patients and health professionals both contribute to decision making by exchanging information and deliberating about available treatment options. SDM is endorsed as the ideal model for treatment decision making in national and international quality standards,^6^, ^7^ supported by evidence that it can improve health outcomes. SDM skills represent a transferable health literacy asset which can support greater autonomy in health decision making situations.

Like general literacy, functional, communicative and critical health literacy can be developed through formal education.^4^ Adult basic education settings are increasingly recognized as an avenue to deliver health content and build health literacy capacity.^8^, ^9^, ^10^ In Australia, existing adult learning infrastructure can be harnessed to capture diverse learner groups including older adults, people living with disabilities, indigenous populations and culturally and linguistically diverse learners.^8^

Health‐education partnerships have shown ability to facilitate meaningful support in health‐related learning for those most in need.^11^ However, SDM has not been incorporated into health literacy programmes for adult learners, and communicative/critical‐level health literacy content has not been evaluated. Including teachers in the evaluation of adult education programs can provide useful insights regarding the learning needs of lower‐literacy learners and the appropriateness of programme content. Qualified teachers have expertise and experience in teaching and reflective practice^12^ which enables them to consciously reflect on their own teaching as well as students’ learning experiences and outcomes. As a key stakeholder, exploring teachers’ perspectives can help to construct meaningful knowledge and understandings and improve the reach and translation of adult education health literacy initiatives.^13^

We developed a novel SDM training programme for adults with lower literacy to develop communicative and critical health literacy skills.^14^ The programme was delivered in Australian adult basic education settings in 2014 by trained adult educators as a core component of a broader health literacy programme and was evaluated as a cluster‐randomized controlled trial.^15^ An exploratory qualitative substudy was conducted with adult educators who participated in the trial to explore views and experiences of teaching health literacy skills (including SDM) and determine whether the programme is contextually appropriate and sufficiently tailored to learner needs, and teaching and institutional objectives.

## 2 METHOD

### 2.1 Intervention: the health literacy programme

The health literacy programme embedded key Learning, Literacy and Numeracy (LLN) skills development into health‐related content.^15^ SDM comprised a 6‐hour core component of the course. Box 1.

Box 1 **SDM programme aims**

**Figure 1:**
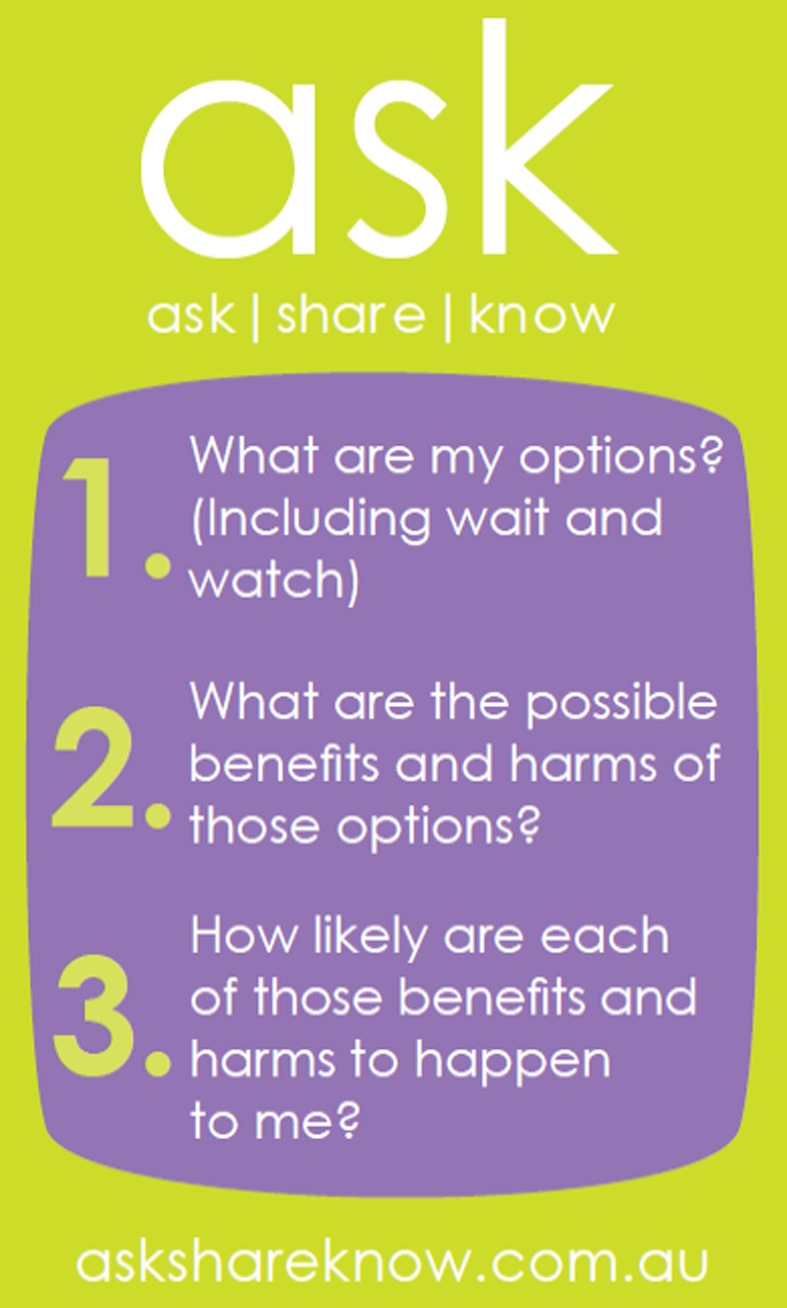
AskShareKnow Student Pocket Card

1 To develop learners’ self‐efficacy and understanding of:

- SDM concepts (including patients’ right to participate) and terminology,
- health risks and benefits including numeric risk information,
- the role of values and preferences in SDM, and
- tools to facilitate SDM (AskShareKnow question‐set^26^; see Figure 1)

### 2.2 Programme delivery and setting

The health literacy programme was delivered by adult educators at Technical and Further Education (TAFE) sites across New South Wales (NSW). TAFE NSW is a government‐funded adult education provider offering low‐cost basic LLN courses. Entry requirements are often flexible, and learners may not have completed secondary schooling. In 2014, over half a million learners were enrolled in TAFE across NSW, including a high proportion of women, unemployed adults, those with a language background other than English, those with low socioeconomic status, mature‐aged learners and learners from regional and remote areas.^16^

All TAFE teachers require a minimal qualification of Certificate IV in Teaching and Assessment. Participating teachers attended a full‐day training course, including a 1‐hour SDM session led by the first author and received a teaching manual with guided lesson plans, learner resources and answers.

### 2.3 Learner population

In total, 167 learners enrolled in basic/beginner LLN courses across regional (33%) and metropolitan (67%) areas of NSW participated in the health literacy programme. The average age of participants was 45 years. The majority were women (69%), spoke a language other than English at home (77%) and had a long‐standing illness or disability (70%). Seventy‐nine percent of participants had inadequate health literacy as measured by the Newest Vital Sign.^17^

### 2.4 Qualitative interview sampling and recruitment

Adult education teachers who delivered the health literacy programme (n=18) were invited to participate^18^ in a 30‐minute interview (face‐to‐face or over‐the‐phone) with an interviewer trained in qualitative methods (DM or SM). Interviews were semi‐structured following a topic guide, but with the flexibility to fully explore participants’ responses. The topic guide included a distinct section on the SDM component of the course (Box 2). Interviews were also conducted with adult learners (n=22) in a similar manner. Ethical approval was obtained from the University of Sydney Human Research Ethics Committee and 10 NSW Institutes of TAFE NSW.

Box 2 **SDM interview topic guide**

1

- Experience of teaching SDM
- Challenges, facilitators and barriers to teaching SDM
- Student reactions, experience and understanding of SDM content
- Thoughts on the application of SDM skills in healthcare settings

Interviews were audio‐recorded, transcribed verbatim and analysed using the Framework approach to qualitative data analysis^19^ (Table 1). This analysis focuses on teachers’ reflections on SDM programme components. Data relating to the other components of the health literacy programme are reported elsewhere.^20^ While the focus of this article is on teachers’ experience of delivering SDM content within a health literacy programme, quotes from adult learners are incorporated throughout to support teachers’ reflections on subjective learner experiences. An in‐depth analysis of learner interviews will be reported elsewhere.

**Table 1:** Data analysis using the five key steps of the Framework approach

Framework steps	Approach
Familiarisation	Three researchers became familiar with the data by independently reading through a selection of the transcripts
Identification of a thematic framework	Researchers independently coded three transcripts before meeting to discuss key themes and constructing an initial coding framework. Using this framework, DM coded a further three transcripts before conferring to discuss, revise and refine the work and generate a final thematic framework
Indexing	The thematic framework was systematically applied to all transcripts. The researchers then organised codes which were valuable for addressing the research question into categories reflecting prominent themes within the data set; (i) cultural and institutional fit (ii) learning experience: a teacher's perspective, (iii) teaching experience, (iv) applying skills beyond the classroom
Charting	A matrix was created for each theme by abstracting, summarising and charting data for each case and each code within that theme
Mapping and interpretation	Thematic analysis was carried out on the managed data set by reviewing the matrices and making connections within and between codes and cases

## 3 RESULTS

Three teachers who delivered the health literacy programme could not be contacted following course completion because they lost their jobs in the state‐wide restructure of TAFE NSW. Interviews were conducted with 15 of 18 teachers. Four interviews with teachers in a job sharing arrangement where the teaching partner delivered SDM content were excluded from this analysis as SDM was not discussed. All 11 teachers included in the final SDM analysis were female. Average teaching experience in adult education was 17 years (Range=1‐34).

We present four themes identified from the data: (i) cultural and institutional fit, (ii) learning experience: a teacher's perspective, (iii) teaching experience, (iv) applying skills beyond the classroom. Quotes included in the text are followed by an identification number, with the letters ‘T’ and ‘L’ used to differentiate teachers’ and learners’ quotes. Learners with the same letter at the end of their ID were enrolled in the same class.

### 3.1 Theme 1: cultural and institutional fit

Shared decision making was perceived by teachers to be an appropriate topic for a basic/beginner adult education context, given learner characteristics and an institutional commitment to learner empowerment.

Teachers described their cohort collectively as a “passive” and “disempowered” group who do not actively participate in decisions about their health. It was the teachers’ view that learners typically deferred decision making to healthcare professionals and accepted test and treatment recommendations without question, reflecting a paternalist, rather than collaborative, model of healthcare decision making. This view was mirrored in adult learners’ own reflections on their participation in past healthcare consultations.

… They're not the sort of people that would ask questions… [they] would just accept what … the doctor says. (HL T1)

When I was younger, you just go there and if he says take this, take that, you walk out the door and go and do it… (HL L11 C)

Teachers saw value in communicative and critical health literacy content in terms of empowering traditionally disadvantaged learners to be more than passive healthcare recipients, with one teacher explicitly stating that, conceptually, SDM matched her teaching philosophy to build learner capacity and her teaching pedagogy of empowerment.

I think it's, it's important for students from a disadvantaged background, whether it be multi‐cultural or, or otherwise, that they try and feel empowered with, with their health especially. (HL T15)

### 3.2 Theme 2: learning experience: a teacher's perspective

#### 3.2.1 New knowledge and main messages

From an educator's perspective, the SDM programme was successful in creating awareness among adult learners of (i) patients’ right to participate in decision making, (ii) question‐asking as a means to participation and (ii) the availability of test/treatment options.

Teachers felt that raising awareness among learners that they could rightfully share in decision making was a powerful contribution of the programme. The SDM programme was perceived to “challenge” learners’ established belief that health professionals are solely responsible for decision making and provide an alternative to their usual passive approach, empowering them to ask questions and engage with providers. This sentiment was echoed in students’ accounts, whereby they reported a new appreciation of the right/responsibility to participate in decision making and increased assertiveness and self‐efficacy for health consultations.

… and they realized that they had rights and that they can actually question what a doctor says, and can be assertive without being rude… they can actually ask questions. (HL T13)

… cos I used to get shy and, like, not say anything… But now I just speak up and then wait for their [healthcare providers’] answer… (HL L20 F)

Teachers felt that learners did not previously engage in a dialogue about treatment alternatives at the point‐of‐care and were constrained by the options presented to them. Training in SDM was thought to generate a new appreciation among adult learners that there can be alternative test and treatment options that can be considered in the light of their benefits and harms. Again, teachers’ reports were consistent with learners’ accounts.

It's made them think that maybe there are choices that they could have…(HL T1)

I know I've got a lot more options… also. But, um, yeah, I know there's definitely options out there now (HL L9 B)

Consideration of benefits and harms was reflected in healthcare experiences learners shared with teachers. In the example below, the teacher associated this learner's recognition and discussion of having experienced a harm with learning about SDM throughout the health literacy course.

… one of the students then would say that they had been taken to the family doctor to be treated for depression, and she was given medication to take and, the medication actually made her feel worse… just the way it was shared made me think that it probably was a result of, um, us talking about shared decision making…(HL T3)

#### 3.2.2 Challenges for adult learners

Teachers perceived there to be challenges for learners throughout the programme, including learning new SDM terminology, understanding the concept of likelihood/risk and interpreting numerical risk presented in different formats.

##### SDM terminology

The language of SDM embedded within the AskShareKnow questions was challenging for adult learners who were unfamiliar with terms such as “options,” “benefits,” “harms” and “likelihood.” Developing understanding took time, particularly for learners from linguistically diverse backgrounds.

I was able to get them to understand. But it took a little bit of work. Basically because of that language. (HL T4)

Teachers felt that activities (e.g. alternative terminology activities) and elements of programme design (e.g. repetitive language) were useful, but also used supplementary activities such as additional vocabulary exercises to reinforce the meanings of particular key terms. Some teachers reported that they replaced words within the AskShareKnow questions with lay terms to support understanding. One learner reported that they had found the teacher's explanation valuable in terms of supporting understanding.

I just had to keep saying good and bad… or problems and no problems, and that sort of… language. (HL T11)

Teacher helps to … explain what, er, what is this word's meaning, and what is this question… then after explaining, every people can understand.(HL L1 A)

##### Interpreting numerical risk estimates

Computational numeracy‐based tasks were perceived as challenging for learners. One teacher commented that understanding numerical risks expressed as percentages was difficult for her cohort, even though the topic was well‐resourced. For others, comparing frequencies with different denominators such as 10 and 100 was noted as a particular challenge.

They did have a little… bit of difficulty understanding the concept that, um… there… if the, if their chance was one out of 10, verses one out of 100… (HL T9)

##### The concept of likelihood

In addition to the challenges of completing computational numerical risk tasks, understanding the concept of individual likelihood/risk embedded within the third AskShareKnow question was noted as a challenge for learners across all campuses. It was difficult for learners to appreciate that, irrespective of the magnitude of objective risk or the frequency of an event in a group of people (e.g. 1 of 100), the individual's probability of being in one risk group (e.g. affected) or the other (e.g. unaffected) is unknown. While some teachers expressed that many learners were beginning to grasp this concept, others felt that comprehensive understanding of “likelihood” would require sustained engagement with the concept beyond the 6‐hour SDM programme.

… particularly the concept of how likely are these benefits and harms to happen to me… the whole concept of probability, certain to very unlikely … I think the activities were excellent, but you could have spent much more time on it. (HL T3)

### 3.3 Theme 3: teaching experience

#### 3.3.1 Past experience and initial perceptions

Teachers had previously taught functional health literacy skills (eg scheduling appointments) within adult basic education. However, no teacher reported having taught SDM.

… I've never really taught that. Because, yes we teach the students to ask questions and how to make appointments… however, we[‘ve] never really gone to that extra step… where they have that shared decision making. (HL T4)

Two teachers expressed that they had been initially sceptical that the SDM content would be too difficult and/or insufficiently engaging for learners in basic/beginner LLN courses. However, after course completion, both felt that adult learners were able to understand and engage with the concept of SDM and communicative/critical health literacy content.

… when I looked at the [SDM] unit I thought, yeah, it looks good but didn't know how they'd cope at all… That unit worked out really well with them and they engaged really well and understood it. (HL T7)

#### 3.3.2 Teaching facilitators

##### Progressive knowledge/skill development

The SDM programme was considered “very clear and well structured,” logically presenting SDM concepts that were new to learners. The AskShareKnow questions provided a logical framework for teaching the multidimensional concept of SDM. Activities and resources focusing on progressive knowledge and skill development were seen by teachers as key facilitators to teaching and learning. This was often discussed with reference to numeracy activities which began with more simple tasks (eg understanding risk labels) and progressed gradually to more complex tasks (eg comparing risks presented as natural frequencies).

It stepped the students through it well, um, so they understood it and seemed to really catch on to that one. (HL T7)

##### Take‐home resources

Teachers felt that learners were enthusiastic about receiving the AskShareKnow pocket card (Figure 1). They valued having a tangible tool to keep after the lesson given that training occurred outside of the healthcare setting. For teachers, the card was used to facilitate discussion and activities including role plays.

And the little card, I have to say, was invaluable. I mean we used it in many contexts, whether they were, you know, in role‐play talking to the chemist, or talking to an acupuncturist, whatever. (HL T10)

##### Supporting social integration

Five teachers reported that the activities within the SDM programme stimulated class discussion and increased integration as learners reflected on content and shared past healthcare experiences.

When we talked a bit more about it, the shyer people that came out later on shared stories… so we could then discuss it and use the things they had talked about earlier. (HL T3)

#### 3.3.3 Teaching challenges

##### Breadth and depth in content coverage

The breadth of content coverage (e.g. right to participate; test/treatment options, benefits and harms; likelihood concepts; numerical probability) and the depth of understanding embedded within the SDM programme made it difficult to teach all programme components within the 6‐hour time‐frame. Teachers felt that lower‐literacy learners would have benefited from additional time for reinforcement and distributed practice of new skills and knowledge. In fact, two teachers did extend the SDM component for this purpose.

It could have been something I would have gone over… So I wished now that I had taught this earlier… so that I could have re‐enforced a lot of that through revision… (HL T11)

### 3.4 Theme 4: applying skills beyond the classroom

#### 3.4.1 Conceptual vs verbatim retention

When asked about the application of SDM knowledge and skills in healthcare settings, most teachers were positive about the potential impact of the programme. Teachers felt that learners were engaged with the concept and empowered to ask questions about treatment options. However, some teachers felt that learners may not use the verbatim wording of the AskShareKnow questions due to the complexity of language. This was consistent with learner interviews in that some students reported asking questions which captured the meaning of the original AskShareKnow questions, simply using alternative terms. For example, many replaced the term harms with “side‐effects” and options with “choices.”

One thing I'll say they might not go in and ask the three questions and that, but I think they would be more empowered to say… hang on a minute, what does that mean for me? (HL T9)

#### 3.4.2 Barriers to behaviour change

Although teachers were generally positive about the programme's potential to facilitate participation, there was acknowledgement that particular learners within basic LLN courses may have difficulty applying the knowledge and skills taught in healthcare encounters due to internal (e.g. intellectual disability) and external (e.g. healthcare provider) factors.

… some I think would. And others probably would have still too many barriers to, um… make that happen. (HL T1)

#### 3.4.3 Cultural norms: a barrier to participation or a teachable moment?

Several teachers commented on “cultural barriers” to SDM, feeling that existing cultural norms may prevent some adult learners from culturally and linguistically diverse backgrounds from engaging in SDM, even after training. Specifically, questioning healthcare professionals was believed to be culturally incompatible with some learners’ views.

But, I think it's still, with quite a few of our students, it's, it's, and probably from a cultural background, that the doctor is, is, is given a position of esteem and you… you certainly wouldn't question a doctor. (HL T7)

In contrast to this perception, however, one teacher reported that learners from a culturally and linguistically diverse campus expressed that it had been useful to learn that patients have the right to participate in healthcare decisions in the Australian healthcare context and that the prevailing medical culture in Australia differed to their previous experience.

So, you know, to find out that you have a choice was very useful for these people, and that was their feedback. (HL T13)

## 4 DISCUSSION

Our study demonstrates that educators see value in incorporating SDM training in adult basic education programmes for communicative and critical health literacy development. SDM training fits the institutional goal of adult education to empower learners and can raise awareness in adults with lower literacy of their right to be involved in healthcare decisions. Our findings suggest that a tailored approach to training, building on foundational skills with language reinforcement and take‐home resources, can facilitate teaching and learning. However, aspects of SDM (likelihood concepts; computational numerical risk tasks; terminology) are challenging for adults with lower literacy. Additional time may be needed to teach all components of the concept and reinforce novel content for meaningful understanding.

Our programme represents the first SDM training programme for adults with lower literacy to be delivered in adult education settings for communicative and critical health literacy skill development. The exploratory design of this qualitative study is a strength as it allowed flexibility to examine the experiences and challenges of teaching a novel SDM programme, as perceived by teachers. Although our sample was small, participating teachers represented institutions across a large geographical area and the socio‐demographic diversity in the study. There was also a wide range of teaching experience within the sample. Adult education teachers are experts in education but are not necessarily health experts. Qualitative evaluation with educators can establish whether training programmes adequately support teachers to deliver content, as well as offer professional insights into curriculum design, delivery and content appropriateness.^9^, ^21^ An earlier qualitative study investigating the impact of a health literacy programme delivered within adult education (but not including SDM) found teachers were concerned about being seen as health experts by learners and that, while the reading level of materials was appropriate, the content was too basic.^22^ This view was not shared by teachers about the SDM content in this study, suggesting that communicative and critical health literacy content can be appropriately designed for groups with lower literacy and can also support adult educators.

Our findings have implications for adult education, research and healthcare practice. First, results reinforce adult education as an appropriate forum to deliver health literacy content, including SDM, to lower‐literacy learners given the fit between content and institutional objectives. Programmes delivered in this setting should be tailored to population needs (e.g. build on foundational skills; incorporate repetitive language), with time to explore concepts and reinforce novel and/or challenging content. Considering that no teachers had taught SDM before, a partnership approach involving both health and education sectors is needed to support the uptake of SDM programmes in this setting,^23^ such that teachers are provided with content and resources while maintaining the flexibility to tailor programmes based on their literacy expertise. There is value in continuing to explore teachers’ perspectives in the evaluation of future programmes to gain insights into both learning and teaching. Additional research should explore novel approaches to developing conceptual risk understanding within programmes of this kind.

Pertaining to practice, our findings reinforce that SDM may be a novel concept for health consumers with lower literacy and from culturally and linguistically diverse backgrounds who may not be aware of their right to participate in healthcare decisions.^24^ This is compounded by language barriers and difficulties understanding numerical risk estimates and probability concepts. Providing all consumers with ‘permission’ to participate and then using accessible language and risk formats (e.g. icon arrays^25^) is necessary to ensure that all consumers can meaningfully engage in healthcare decision making. Community‐based programmes, such as our health literacy programme, must be complimented by a healthcare environment which is supportive of, and responsive to, lower‐literacy needs.

## 5 CONCLUSION

Adult basic education is rooted in a historical context that emphasizes learner empowerment. SDM training for critical and communicative health literacy develops a transferable skill‐set which can support autonomy in a range of settings and situations; an outcome which wholly aligns with the culture and values of adult education. Teachers are positive about teaching SDM skills within health literacy programmes delivered in this setting, and our study suggests that programmes can be designed in a way that both supports teachers to deliver novel SDM content and empowers learners. There is benefit in fostering collaboration between adult education and healthcare sectors to reach disadvantaged populations who are rarely the focus of SDM interventions, but who stand to benefit the most from them.

## CONFLICT OF INTERESTS

None to declare.
